# Insanity and Temperament. I.—Mediæval *v*. Modern Ideas in Medicine

**Published:** 1916-05-06

**Authors:** 


### INSANITY AND TEMPERAMENT.

### I. Mediæval v. Modern Ideas in Medicine.

The word "temperament" was much used by our forefathers, and had for them a definite and important meaning, and the thing it stands for was much considered and studied by them, and formed an important consideration in their practice of medicine. The word is little used nowadays, nor is the thing it stands for much regarded, and we are apt to scoff and sneer at our forefathers for using it at all. A generation ago, unless a thing could be weighed in bulk, seen under the microscope, or identified by chemical reaction, we were inclined to deny its existence. This is perhaps a better attitude than that of general and indiscriminate credulity, but it is apt to result in narrowness of view, and in blindness to important matters that cannot be submitted to physical or chemical tests. That wave has passed on, and we are now in the trough between it and the next. Now we are all devotees of hypnotism, telepathy, psycho-analysis, Christian Science, suggestion, or psycho-therapy in some form or other; and our faith in these things is certainly neither wanting in robustness nor proportioned to the tenuity of the results that accrue from them. Vain and useless as they are for the most part, and pernicious as they are in large part, these methods have at any rate this merit—that they do recognise the importance of things that are not material things. They do recognise the desirability of taking into consideration, in the treatment of disease, the mental constitution as well as the physical constitution of the patient.

### Surgeon, Physician, and Patient.

The besetting vice of surgery is that it is apt to look upon disease as affecting a single organ, and assuming that if that organ is removed the disease will disappear, and the patient will recover. The disease of the organ is removed with the organ, the wound heals, and the surgeon triumphs and departs; occasionally the patient dies of the rest of his malady which was unaffected by the operation. The view taken by the physician is less limited, but it is very apt to be incomplete. He regards the patient as a very complicated mechanism which has fallen out of gear in this respect and in that, and his business is to discover the source of the derangement and to rectify it as far as possible. When the physical apparatus is again in working order he, too, departs, and considers his patient cured. But very often the patient is not then cured. His physical apparatus may be working satisfactorily, but yet there is something wrong. He cannot do his work satisfactorily. He is not happy. His relations with his wife and family are not what they ought to be. There is a hitch somewhere: there is some weakness, something awry, something that has not been taken into consideration, because the patient has been looked upon as an aggregate of organs and tissues performing functions, and not as a man, not as a whole, not as an integrated living being, living by virtue of his relations and his intercourse with the world around him. The physician cannot treat his patient satisfactorily unless he looks upon his patient, not as a complicated bundle of organs, one or more of which is deranged in its function, but as a whole—as a man having relations with the world around him, on which he acts and which reacts on him; and with respect to these actions and reactions the man is summed up, not in his organs and their functions, but in his mind.

Disease cannot be rightly, comprehensively, or satisfactorily treated unless the treatment is directed not to the disease, but to the man who is suffering from the disease, to the whole man, who may be described as a mind working through and by means of his body. By all means rectify the bodily functions; but do not think that when you have relieved the patient of his bodily disease you have necessarily cured the patient. That is not done until he is restored to efficiency in dealing with his circumstances, and this is a matter that concerns primarily his mind. Patients used to have faith in the old family doctor because he "understood their constitutions." Modern scientific physicians, especially when they are young, are apt to smile at the phrase, and to consider it an antiquated superstition, but there was much truth in it. The family doctor, who had brought both the patient and perhaps the patient's future wife also into the world; had attended them in their childish ailments, talked over them with their parents, seen them off to school and welcomed them home again, advised about their occupations and watched their careers, accumulated a store of knowledge about them that was invaluable when his services were needed. He knew their weak points and their strong points, both bodily and mental. He knew what they could do and what they could not. He knew how they were likely to react, not only to drugs, but to prosperity and adversity, to kindness and severity. In short, he knew their physique, and he knew their temperament.

### Physique and Temperament.

The physique, or, as the mediæval physicians called it, the corporature, is the bodily constitution of the patient, the order of architecture, as it were, to which his body belongs, the way in which he is compounded; and so the temperament is his mental constitution, the order of mental architecture to which his mind conforms, the way in which his mind is put together, the absolute and relative magnitude of its ingredients, and the general nature of its structure. The temperament is the physique or corporature of the mind, another word as well as the idea come down to us from a remote past. Ancient and mediæval medicine was dominated by two theories, which fitted together and reinforced one another. The first of these was Astrology, the supposed influence of the planets on human lives; the second was the humoral pathology, which ascribed all disease to the excess or defect of one of the four humours—yellow bile or choler, black bile, blood, and phlegm. According to the first of these theories, not only was every human being under the jurisdiction of one or more of the planets, but also he showed in his physical constitution or corporature—his physique as we should now call it—to which of the planets he was subject, so that mankind were classifiable on sight into the Jovial, the Saturnine, the Martial, the Mercurial, the Venereal, the Lunar or Lunatic, and the Solar; for the sun and moon were then included among the planets. Each of these physical constitutions was accompanied by a corresponding mental constitution, and it is to these mental constitutions or temperaments that we now refer when we speak, as indeed we seldom do, of a man as a jovial, or martial, or saturnine, or mercurial person, or a lunatic. The jurisdiction of the planets over human dispositions was partly direct and was partly exercised through the humours, over which also the planets extended their sway. The humours abounded or were defective according to the power of the planet that ruled them was powerful or the reverse, so that jovial and solar persons, in whom blood predominated, were sanguine; saturnine persons were bilious or, strictly speaking, atrabilious; martial persons, in whom choler or yellow bile abounded, were choleric; and venereal persons and lunatics phlegmatic. For some reason that cannot now be traced we have ceased to speak of venereal and solar persons, but we retain in common speech all the other terms, though we retain them with a grip that is much relaxed, and is still relaxing, and as far as we do retain them we use them in meanings departing a good deal from their original significations. What meaning they still retain, moreover, is limited strictly to the mental constitution or temperament, so that when we now use a term belonging to either set, as, for instance, jovial or sanguine, martial or choleric, we characterise by it a certain bent or constitution, or disposition of mind and not of body. By a sanguine person we mean no more than a person of hopeful, optimistic disposition; by a jovial person no more than a jolly, smiling, good-humoured, expansive person; by each astrological or humoral term we mean one mental quality and no more; but it was not thus that the names were originally bestowed. To our ancestors a temperament signified the total mental constitution of a man, just as by physique we mean his total bodily constitution. The temperament meant no single quality, but the whole construction of the mind; and the significance of the astrological or humoral title was that it assumed certain specific combinations of mental qualities, it assumed that the mental qualities occur in certain sets, which are met with again and again in different people, of course with individual variations, but substantially true to type. Is there any ground for the supposition that mental qualities do, in fact, thus occur in groups specifically alike? We must remember that this is a matter that requires no microscope or spectroscope, no knowledge of chemistry or physiology to determine. It is to be determined by the mere observation of human nature as exhibited in individual men and women, and for this observation our forefathers were just as well equipped as we are. For such observations of human nature the ancient and mediæval physicians possessed every facility that we possess now, and they were not distracted by having to observe a great multitude of things that we must observe. What they have left on record shows that in many things they were very shrewd and acute observers, and although we cannot accept the astrological and humoral theories that they invented to account for what they observed, we need not on that account reject their observations. We need not empty the baby out with the bath water. The evidence of these latter days goes far to confirm the observation of the ancient and mediæval physicians, that mental qualities do tend to occur in groups, that there are different temperaments, some very distinct, marking their possessors off from the rest of mankind, others less distinct; some constituted by a cluster of many qualities, others by few; and that though a temperament is not always present in its purity, or in high degree, yet when it is so present the individuals who possess it present a likeness in mental characters to one another such as is rarely presented by the different members of a single family. In subsequent articles we shall describe certain specific temperaments.

